# Acute Pancreatitis Due to Clipping of the Ampulla With Over-The-Scope Clip as a Complication of Bleeding Duodenal Ulcer Treatment

**DOI:** 10.7759/cureus.8963

**Published:** 2020-07-02

**Authors:** Gurbir Sehmbey, Indu Srinivasan, Keng-Yu Chuang

**Affiliations:** 1 Internal Medicine, Banner University Medical Center, Phoenix, USA; 2 Internal Medicine, University of Arizona College of Medicine - Phoenix, Phoenix, USA; 3 Gastroenterology, Valleywise Health Medical Center, Phoenix, USA; 4 Internal Medicine/Gastroenterology, Valleywise Health Medical Center, Phoenix, USA; 5 Internal Medicine/Gastroenterology, Creighton University School of Medicine-Phoenix Program, Phoenix, USA

**Keywords:** pancreatitis causes, endoscopic approach, over-the-scope clips, peptic ulcer, gi endoscopy

## Abstract

Over-the-scope clips (OTSC) (Ovesco, Tübingen, Germany) are commonly used for closure of bowel perforations, fistulas and to achieve hemostasis. This device is attached to the endoscope and delivers a variety of clips, based on diameter and depth, that works through tissue approximation. Complications including local inflammation, ulcers, or obstruction can occur. When the clip is misplaced or OTSC-associated complications occur, OTSC removal may be indicated. We present a case of a patient who presented to our hospital with upper gastrointestinal (GI) bleeding. OTSC was used to achieve hemostasis, however, the clip was misplaced over the ampulla of Vater. remOVE system (Ovesco, Tübingen, Germany) was used to remove the misplaced clip.

## Introduction

Over-the-scope clips (OTSC) (Ovesco, Tübingen, Germany) are extensively used for the management of gastrointestinal (GI) bleeding, stent anchoring, fistulas, and bowel perforations [1]. OTSC comprises a variety of clips based on the diameter and depth of OTSC caps. It can also be categorized using the type of teeth: atraumatic with blunt teeth or traumatic with sharp teeth [2]. Its mechanism of action includes mechanical compression and tissue approximation [3]. Although designed to be a durable implant, made up of biocompatible nitinol material, OTSC removal is indicated in certain situations [4]. Removal of OTSC is indicated when complications occur, which include local inflammation, ulceration, luminal obstruction, or clip misplacement [5]. remOVE system (Ovesco, Tübingen, Germany) is a safe and effective tool designed to remove the clip.

## Case presentation

A 56-year-old male with a history of hereditary hemochromatosis, heroin, and alcohol abuse presented to our hospital with abdominal pain and melena for four days. The patient's vital signs were stable on arrival. On examination, the patient had moderate epigastric tenderness with positive bowel sounds. Rectal exam revealed melanotic stool with bright red streaking, no mass or lesions were felt. Laboratory evaluation revealed WBC 15.8 k/mm^3^, Hgb 10.4 g/dL and lipase 321 U/L. CT abdomen/pelvis revealed active bleeding in the 2nd and 3rd portion of the duodenum along with numerous enlarged vessels within the mucosa. The patient was started on intravenous pantoprazole and packed red blood cell infusion was initiated.

**Figure 1:**
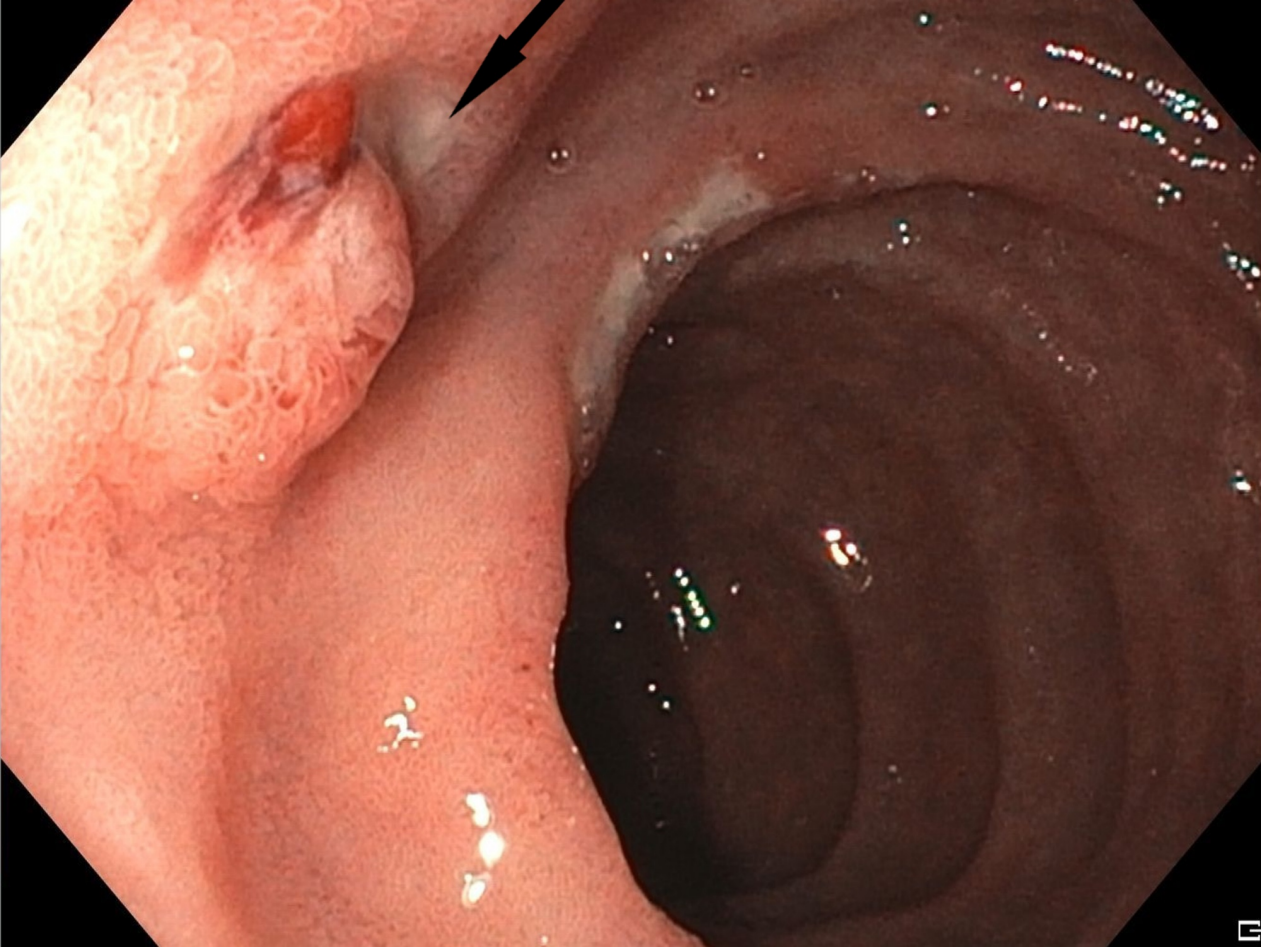
EGD showing a peptic ulcer with an exposed vessel in the 2nd portion of duodenum proximal to the ampulla EGD: Esophagogastroduodenoscopy

Esophagogastroduodenoscopy (EGD) was performed which revealed a peptic ulcer with an exposed vessel in the 2nd portion of duodenum proximal to the ampulla (Figure 1). Hemostasis was achieved using epinephrine injection and placement of an OTSC. The patient was admitted to the hospital for observation. Overnight, the patient’s hemoglobin stabilized, however, he developed excruciating sharp epigastric pain radiating to the back. The abdominal X-ray did not reveal any acute etiology. Serum lipase was checked and was elevated to 17,925 U/L, liver function tests were normal. The patient was treated supportively overnight with bowel rest, intravenous fluids, and pain medications.

**Figure 2:**
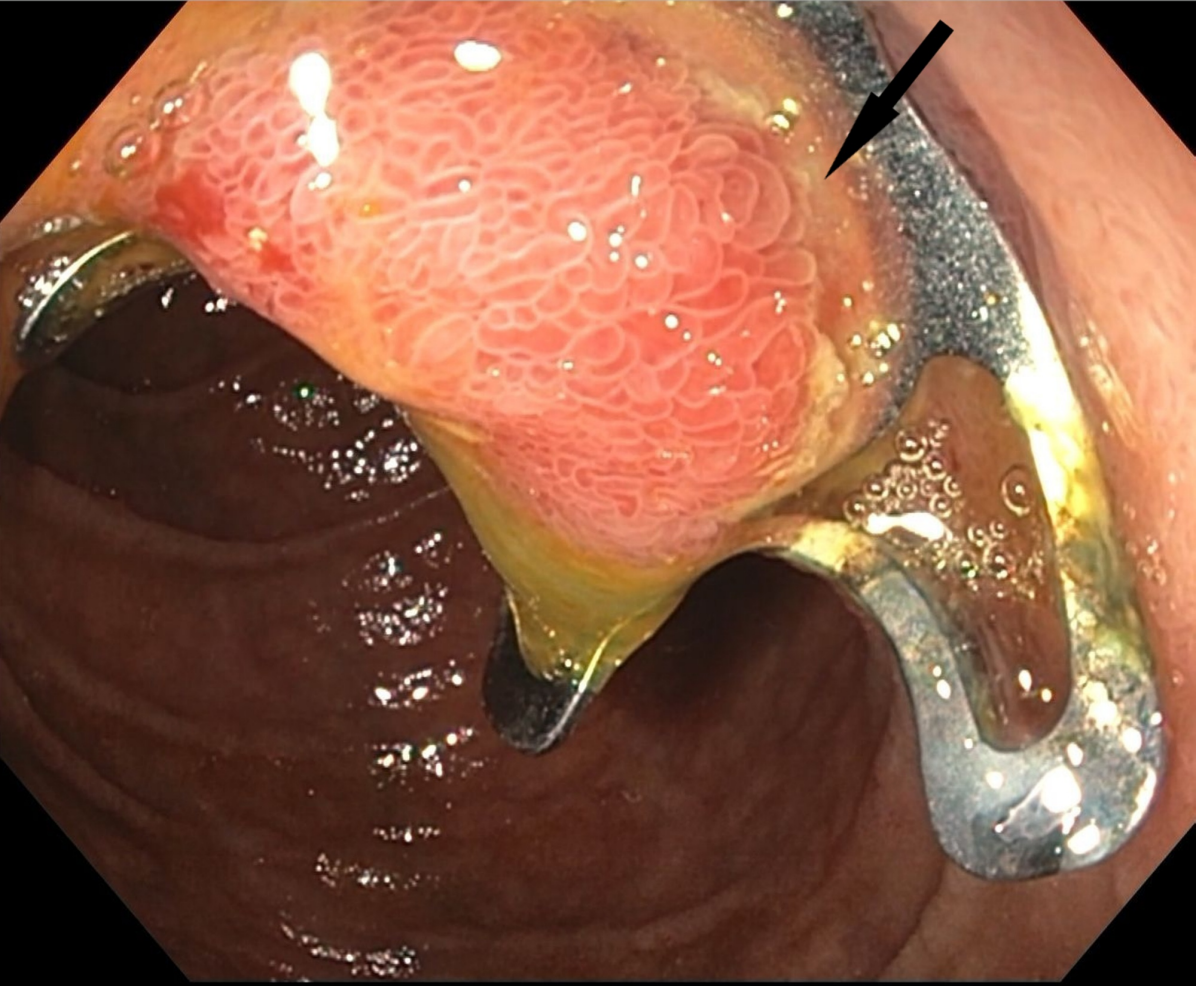
Repeat EGD showing clip deployed over the ampulla EGD: Esophagogastroduodenoscopy

The next day, magnetic resonance cholangiopancreatography (MRCP) was performed which revealed diffuse pancreatic edema, dilated pancreatic duct measuring 5 mm, and OTSC located in the medial 2nd portion of the duodenum, in the region of the ampulla. A repeat EGD performed showed OTSC deployed over the ampulla (Figure 2). Attempts to break the clip using a needle knife and argon plasma coagulation (APC) were unsuccessful. The patient was treated supportively for acute pancreatitis.

**Figure 3:**
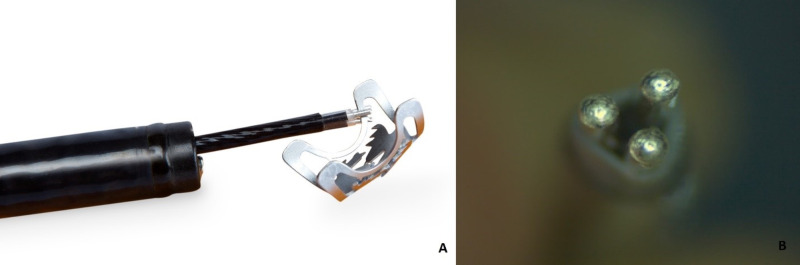
DC Cutter: A) grasping the frame of an OTSC, B) tip consisting of three electrodes (with permission from Ovesco, Tübingen, Germany) OTSC: Over-the-scope clip; DC: Direct current.

**Figure 4:**
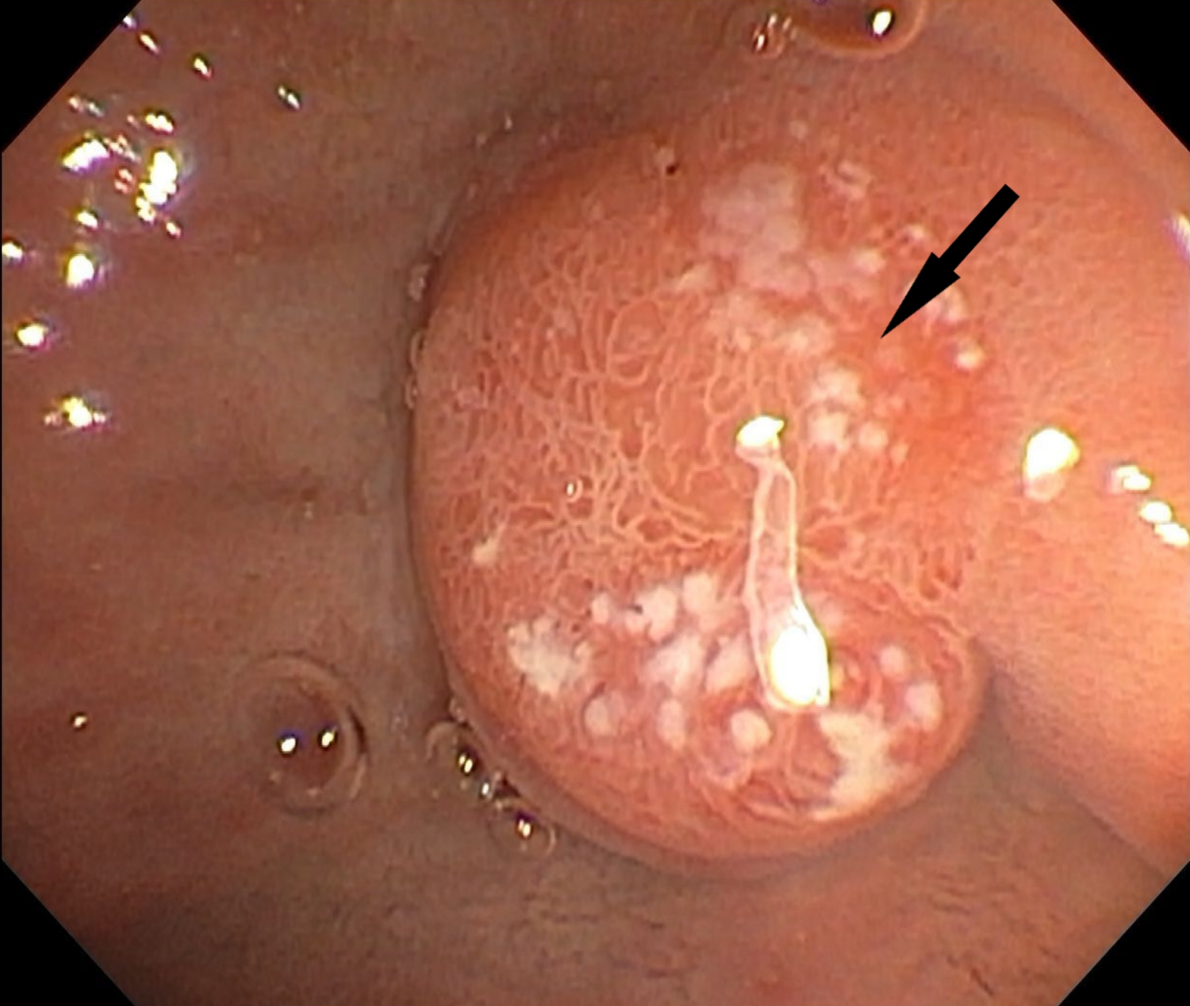
Repeat EGD one month later showed normal duodenum with healed ampulla and ulcer EGD: Esophagogastroduodenoscopy

Two days later remOVE system was obtained. A repeat EGD was performed and using the bipolar direct current (DC) cutter (remOVE system) (Figure 3) the clip was removed and extracted, following which bile was seen to be draining freely from the ampulla. Serum lipase trended down and the patient experienced an improvement in abdominal pain. The patient was discharged the next day. Repeat EGD one month later showed normal duodenum with healed ampulla and ulcer (Figure 4).

The abstract of this article has been presented at the 'American College of Gastroenterology Conference' in 2019 [6].

## Discussion

OTSC is a reliable and effective clipping system intended for endoscopic treatment of acute GI bleeding, full-thickness wall closures, and management of complications after endoscopic and surgical interventions [7]. The OTSC for flexible endoscopy is an elastic device that works by compression and approximation of tissue in the digestive tract [8]. OTSCs are durable, composed of Nitinol (nickel-titanium alloy), a biocompatible, and MR conditional material that can remain in the body as a long-term implant (Ovesco Endoscopy, Tübingen, Germany). OTSC-associated complication rate is 9.5% [9]. Removal of OTSC may be indicated when complications (ulceration, inflammation, and luminal obstruction) or clip misplacement occurs [9].

Numerous methods of OTSC removal have been described, including the use of APC, Nd: YAG laser, or cooling of the clip using ice-cold saline to deform the clip structure [10,11]. The extraction of the clip is done in a closed position and with the use of guidewire [12]. OTSC removal may be complicated with ingrowth into the GI wall depending on the length of time the OTSC is in place [13]. This may require debridement of granulation tissue prior to clip removal. Removal of OTSC may also be difficult depending on the site of OTSC. Sites within the GI tract such as duodenal knee and colonic flexures may limit the mobility of the endoscope and make it difficult to grasp and cut the OTSC [13]. After clip removal, minor bleeding can be seen at the former OTSC site [9].

remOVE system can be used for endoscopic OTSC clips removal from the GI tract. It includes DC Cutter which is a bipolar instrument that allows fragmentation by localized melting and cutting of OTSC when used with DC Impulse. The DC Cutter’s tip consists of three electrodes that are brought in contact with the thinnest parts of the nitinol clip which is the frame/hinge. The application of DC impulse heats up and melts the nitinol. The OTSC must be cut at two spots on the opposite side of the row of teeth and the clip fragments can be extracted. Overall, the remOVE system has demonstrated safety and effectiveness for clip removal with a success rate of 92.9% in the upper and lower GI tract [13].

## Conclusions

Endoscopic treatment is very effective in the treatment of upper gastrointestinal bleeding (GIB). In cases of recurrent peptic ulcer bleeding after successful initial hemostasis, OTSC is superior to the standard therapy. OTSC removal is indicated if clip-associated complications occur. remOVE system is a novel bipolar cutting device which is effective and safe in clip removal.
